# Closing the gap: endoscopic treatment of esophageal anastomotic leakage—a retrospective cohort study

**DOI:** 10.1007/s00464-025-11904-0

**Published:** 2025-07-14

**Authors:** Myriam W. Heilani, Daniel Teubner, Thomas Haist, Mate Knabe, Patrizia Malkomes, Florian Alexander Michael, Michael Stumpf, Stefan Zeuzem, Wolf Otto Bechstein, Mireen Friedrich-Rust, Georg Dultz

**Affiliations:** 1 Medical Clinic 1, University Hospital, Goethe University Frankfurt, Frankfurt am Main, Germany; 2 Helios Dr. Horst Schmidt Kliniken, Wiesbaden, Germany; 3 Asklepios Paulinenklinik Wiesbaden, Wiesbaden, Germany; 4 Centrum Gastroenterology Bethanien, Bethanien-Hospital, Frankfurt am Main, Germany; 5 Department of Surgery, Knappschaft Kliniken University Hospital Bochum, Ruhr-University Bochum, Bochum, Germany; 6 Department of General, Visceral, Transplant and Thoracic Surgery, University Hospital, Goethe University Frankfurt, Frankfurt am Main, Germany

**Keywords:** Anastomotic leakage, Esophageal defects, Endoscopic vacuum therapy, Endoscopic vacuum-assisted closure, Esosponge, SEMS

## Abstract

**Background:**

A variety of endoscopic techniques are available for the closure of esophageal defects, each offering distinct advantages. Endoscopic vacuum therapy (EVT) has emerged as a highly effective approach. Alternatively, fully covered self-expanding metal stents (SEMS) can be placed intraluminally until defect closure is achieved. For smaller defects, over-the-scope clips (OTSC®) provide a viable option. However, comparative data remain limited and reported closure rates vary widely across the literature. This study aimed to evaluate and compare closure rates of postoperative anastomotic leaks treated with EVT, SEMS, and OTSC® at two specialized centers in Germany.

**Methods:**

This retrospective study included all patients treated endoscopically for anastomotic leakage at two tertiary gastroenterological centers between May 2007 and February 2023. The primary endpoint was successful endoscopic defect closure. Secondary endpoints included in-hospital mortality, need for revision surgery, duration of therapy, hospital stay length, time to treatment initiation, and procedure-related complications.

**Results:**

A total of 59 patients (71% male, mean age 64 years) were included. In 94.9% of cases, surgery was performed for oncologic indications; 46% had received neoadjuvant therapy. The most common procedure was Ivor Lewis esophagectomy (89.8%). EVT was used in 24 patients, SEMS in 32, and OTSC® in 14. The overall closure rate was 78.0%. Patients with successful closure had a significantly lower ASA score (*p* = 0.039), smaller defects (≤ 1 cm: 57.8% vs. 15.4%, *p* = 0.007), lower in-hospital mortality (2.2% vs. 38.5%, *p* < 0.001), and reduced need for revision surgery (0% vs. 61.5%, *p* < 0.001).

**Conclusions:**

The success of endoscopic therapy is closely linked to patient health status and defect size. Notably, esophageal defects ≤ 1 cm can almost always be closed successfully using endoscopic methods.

**Graphical abstract:**

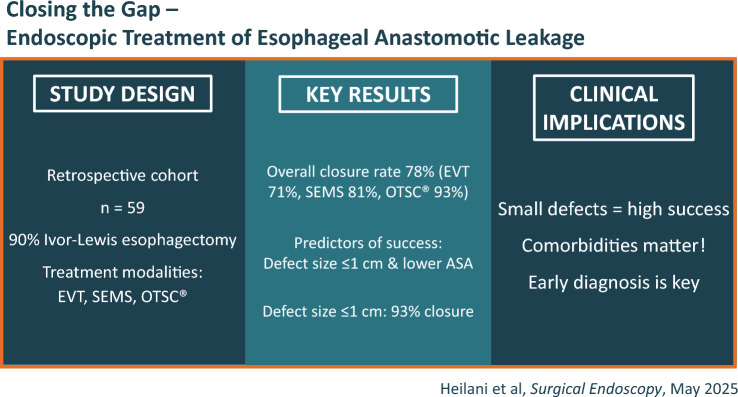

Optimal management of transmural esophageal defects remains an interdisciplinary challenge and the subject of current clinical research [1]. These defects can arise from either traumatic or oncologic causes, each requiring distinct therapeutic approaches. Traumatic esophageal injuries may result from external trauma, severe vomiting (Boerhaave syndrome), or iatrogenic damage during endoscopic or surgical interventions. In contrast, oncologic esophageal defects most commonly occur as postoperative complications following esophagectomy for esophageal malignancy. Transthoracic esophagectomy remains the standard surgical treatment for esophageal cancer but it is associated with a significant risk of morbidity and mortality with postoperative anastomotic leakage occurring in 11.4–21.2% of cases [2, 3].

Depending on the size of the defect, various endoscopic treatment modalities can be considered for closure. EVT is now regarded one of the standard techniques for managing transmural esophageal defects [4]. The concept of vacuum therapy is well established in secondary wound healing of surgical patients. It actively absorbs wound secretions, promotes blood circulation, and stimulates the formation of granulation tissue in the wound [5]. The concept has been translated to an endoluminal application, with the first reported endoscopic placement in the esophagus dating back to 2008 [4]. The procedure is carried out as follows: A transnasal draining tube connected to a polyurethane sponge at its distal tip is inserted into the defect, either intraluminally (in front of the defect) or intracavitary (into the defect itself). Once in place, a continuous negative pressure is applied via the tube. The sponges are replaced every few days until complete closure of the defect. Endoscopic vacuum therapy is an established treatment option for esophageal defects, especially in cases of postoperative anastomotic leakage. The largest studies published to date have reported closure rates of 78–93% [6–9].

Other options for closing esophageal defects include the use of SEMS or OTSC®. However, the latter are only suitable for closing defects up to a maximum of 2 cm [10]. SEMS represent the most used treatment alternative to EVT. They have proven to be effective, covering the defect intraluminally and remaining in situ until complete healing. Reported closure rates range from 70 to 81% [9, 11, 12]. However, being placed intraluminally they do not drain the cavity behind the defect which is a disadvantage that has to be considered while choosing the adequate treatment option. Recently, a novel VAC-Stent (MICRO-TECH Europe GmbH, Düsseldorf, Germany) has been introduced, combining the principles of a fully covered stent with negative pressure therapy. Using the VAC-Stent, Lange et al. reported a closure rate of 80% in 15 patients, with treatment lasting an average of 15 days and requiring a median of 2 stents per patient [13].

In the present study, we aimed to analyze the closure rates of postoperative anastomotic leakage treated with EVT using a sponge, SEMS and OTSC® in two German gastroenterological tertiary centers. As closure rates vary significantly across studies, we also aimed to identify potential risk factors for unfavorable outcomes.

## Materials and methods

### Study design

Two German gastroenterological tertiary centers participated in this retrospective cohort study (University Hospital Frankfurt and Helios Dr. Horst Schmidt Hospital Wiesbaden). We scanned medical records using appropriate ICD and OPS Codes to identify all patients with esophageal defects undergoing endoscopic treatment. Relevant demographic and clinical data were retrieved. Data are displayed following STROBE reporting standard.

We only included patients with postoperative anastomotic leakage as the cause of the defect, while patients with spontaneous rupture/Boerhaave syndrome were excluded. Additionally, only therapeutic endoscopic treatment by sponge, stent or OTSC® Clip was included, whereas patients who received prophylactic sponge or stent treatment were excluded.

Primary endpoint was successful endoscopic closure of the defect. Secondary endpoints included defect size, number of stent or sponge exchanges, length of endoscopic therapy, length of hospital stay, in-hospital mortality, and complications (major bleeding, strictures, sponge erosion, stent migration).

The suspected diagnosis of an esophageal leak was based on clinical findings, including fever, rising inflammatory markers, and changes in the composition of drainage fluids. In cases of concrete suspicion, upper endoscopy (EGD) was performed the same day for confirmation. If a leak was identified, treatment with either a stent, endoscopic vacuum therapy (sponge), or an OTSC® clip was initiated during the same procedure. When EVT was used, intracavitary placement was performed whenever it was feasible. Otherwise, the sponge was placed intraluminally. In all patients receiving EVT, a negative pressure of 125 mmHg was applied by connecting the tube to an electronic vacuum device. The sponge was exchanged every 3–4 days, based on the decision of the treating endoscopist. The sponge system was either created individually in cooperation with the treating general surgeon using a nasogastric tube, polyurethane sponge, and suture material or using the Eso-SPONGE® system (B. Braun SE, Melsungen, Germany). For stent treatment different manufacturers of fully covered esophageal stents were used. No VAC Stents were employed in this study. Clips used in this study were OTSC® Clips and OTSC® Stentfix (Ovesco Endoscopy AG, Tübingen, Germany). The final decision regarding the type of endoscopic treatment was made by the respective endoscopist in collaboration with the treating surgeon.

The study was performed in accordance with the Declaration of Helsinki. The local ethics committee approved this study (No: 2023-1313).

### Statistical analysis

Data were collected using Excel Version 16.91, Microsoft Inc., Redmond, USA. Statistical analyses were performed using SPSS v18.0; SPSS Inc., Chicago, Ill, USA. For patients’ characteristics, group differences were assessed by Chi-Square for non-normally distributed nominal variables, *t* test for normally distributed nominal variables, Mann–Whitney U for ordinal and metric variables and Kruskal–Wallis Test for UICC classification. SPSS was also used for a subsequent binary logistic regression model (univariate and multivariate). A *p* value of less than 0.05 was considered significant. Cases with missing data in relevant variables were excluded from the analysis (listwise deletion).

## Results

### Overall patient characteristics

Overall, 68 patients were identified for study inclusion. Two patients with Boerhaave syndrome as cause of the esophageal defect were excluded. Seven patients who solely received prophylactic sponge placement were also excluded. A total of 59 patients undergoing endoscopic treatment between May 2007 and February 2023, all with postoperative anastomotic leakage as cause of the defect, were included in this study. Patients’ characteristics are displayed in Table 1.

**Table 1:** Patient characteristics of the overall cohort and the subgroup of patients with and without endoscopic treatment success

Patients’ characteristics	All patients (*n* = 59)	Patients with endoscopic treatment success (*n* = 46)	Patients without endoscopic treatment success (*n* = 13)	*p*^§^
Age, y, mean (SD)	64 (8.8)	64 (8.9)	64 (8.9)	0.335
Male sex, *n* (%)	42 (71.2)	35 (76.1)	7 (53.8)	0.118
ASA Score				**0.039**
I, *n* (%)	1 (1.7)	1 (2.2)	0 (0.0)	
II, *n* (%)	30 (50.8)	26 (56.5)	4 (30.8)	
III, *n* (%)	25 (42.4)	18 (39.1)	7 (53.8)	
IV, *n* (%)	2 (3.4)	1 (2.2)	1 (7.7)	
V, *n* (%)	1 (1.7)	0 (0.0)	1 (7.7)	
Diabetes mellitus, *n* (%)	14 (23.7)	11 (24.4)	3 (25.0)	0.968
Obesity, *n* (%)	10 (21.3)	8 (17.8)	2 (16.7)	0.928
Smoking, *n* (%)	29 (55.8)	23 (56.1)	6 (54.5)	0.927
Etiology of esophageal defect, *n* (%)				
Postoperative anastomotic leakage	59 (100)	46 (100)	13 (100)	
Indication for surgery before endoscopic treatment				0.628
Oncologic resection, *n* (%)	56 (94.9)	44 (95.7)	12 (92.3)	
Histology: SSC, *n* (%)	13 (23.2)	11 (23.9)	2 (16.7)	
Histology: ACA, *n* (%)	43 (79.6)	33 (71.7)	10 (83.3)	
Revision surgery after fundoplication, *n* (%)	3 (5.1)	2 (4.3)	1 (7.7)	
UICC classification				0.361
IA, *n* (%)	10 (20.0)	10 (25.0)	0 (0.0)	
IB, *n* (%)	10 (20.0)	9 (22.5)	1 (10.0)	
IIA, *n* (%)	11 (22.0)	5 12.5)	6 (60.0)	
IIB, *n* (%)	9 (18.0)	7 (17.5)	2 (20.0)	
IIIA, *n* (%)	0 (0.0)	0 (0.0)	0 (0.0)	
IIIB, *n* (%)	6 (12.0)	6 (15.0)	0 (0.0)	
IIIC, *n* (%)	4 (8.0)	3 (7.5)	1 (10.0)	
Neoadjuvant therapy, *n* (%)	33 (62.3)	25 (58.1)	8 (80.0)	0.199
Radiochemotherapy, *n* (%)	10 (18.9)	8 (18.6)	2 (20)	0.403
Chemotherapy, *n* (%)	23 (43.4)	17 (39.5)	3 (60)	
None, *n* (%)	20 (37.7)	18 (41.9)	2 (20)	
Surgical approach				
Abdomino-thoracic, *n* (%)	53 (89.8)	41 (89.1)	12 (92.3)	0.490
Abdomino-thoracic-cervical, *n* (%)	4 (6.8)	4 (8.7)	0 (0.0)	
Transhiatal extended gastrectomy, *n* (%)	1 (1.7)	1 (2.2)	0 (0.0)	
Other^$^, *n* (%)	1 (1.7)^$^	0 (0.0)	1 (7.7)^$^	
Reconstruction/ type of anastomosis				0.218
Esophagogastrostomy, *n* (%)	51 (86.4)	41 (89.1)	10 (76.9)	
Esophagojejunostomy, *n* (%)	6 (10.2)	4 (8.7)	2 (15.4)	
Colonic interposition, *n* (%)	1 (1.7)	1 (2.2)	0 (0.0)	
Residual tumor status (R-status) after resection				0.624
R0, *n* (%)	45 (91.8)	38 (92.7)	7 (87.5)	
R1, *n* (%)	4 (8.2)	3 (7.3)	1 (12.5)	
Location of the defect, cm, median (range)	25 (17–40)	25 (17–40)	28 (20–40)	
Proximal (≤ 20 cm), *n* (%)	5 (9.8)	4 (10.3)	1 (8.3)	0.588
Middle third (> 20 cm), *n* (%)	40 (78.4)	31 (79.5)	9 (75.0)	
Distal third (> 30 cm), *n* (%)	6 (11.8)	4 (10.3)	2 (16.5)	
Defect size, cm, median (range)	1.5 (0.3–10)	1 (0.3–10)	2 (0.5–6)	0.127
Defect size ≤ 1 cm, *n* (%)	28 (48.3)	26 (57.8)	2 (15.4)	**0.007**
Defect size > 1 cm, *n* (%)	30 (51.7)	19 (42.2)	11 (84.6)	
Defect size ≥ 3 cm, *n* (%)	17 (29.3)	13 (28.9)	4 (30.8)	0.896

Bold values are statistically significant (*p* < 0.05)

^$^Revision surgery after complicative laparoscopic fundoplication

^§^Using Chi-Square for non-normally distributed nominal variables, *t* test for normally distributed nominal variables, Mann–Whitney *U* for ordinal and metric variables and Kruskal–Wallis Test for UICC classification

Mean age was 64 years (SD 8.8) and 71.2% of patients were male. The ASA Score was either II (50.8%) or III (42.5%) in most cases. The Indication for surgery was oncologic resection (56/59) and revision surgery after complicated fundoplication (3/59). The UICC classification was either I (40.0%) or II (40.0%) in most cases. Of those undergoing oncologic resection, 57% received neoadjuvant therapy (radiochemotherapy in 16.9% of cases and chemotherapy in 40% of cases). The surgical approach was abdomino-thoracic in most cases (89.8%) with Ivor Lewis resection being the most common reconstruction (86.4%). The defect was located at a median of 25 cm (range 17–40) from the alveolar margin, with most defects located in the “middle third” (78.4% located > 20 to 30 cm from alveolar margin). Residual tumor status after resection was R0 in most cases (91.8%). Median defect size was 1.5 cm (range 0.3–10) with 48.3% of defects being ≤ 1 cm, 51.7% > 1 cm and 29.3% ≥ 3 cm.

**Fig. 1:**
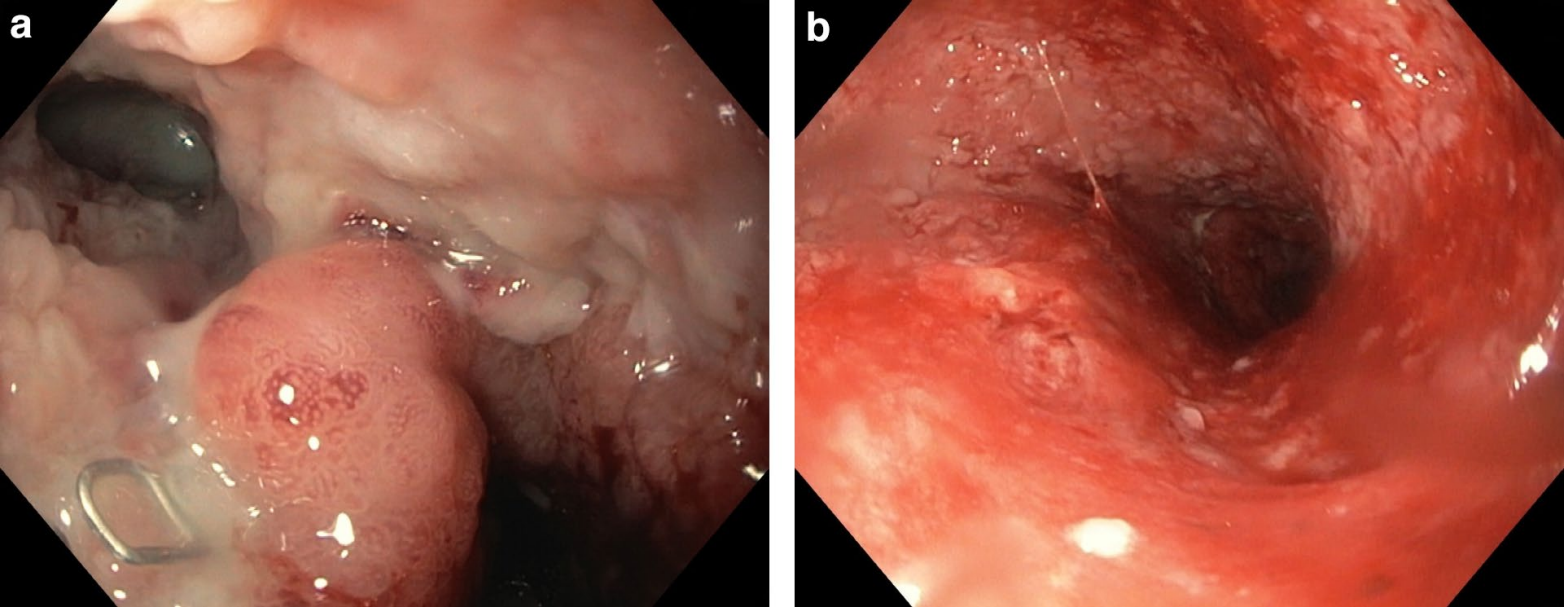
**a** Anastomotic leakage at 10 o’clock position after esophagectomy with subsequent intracavitary placement of the sponge. Visible surgical staple in the lower left quadrant of the image. **b** Result after four sponge changes with closed leakage and visible granulation of tissue

**Fig. 2:**
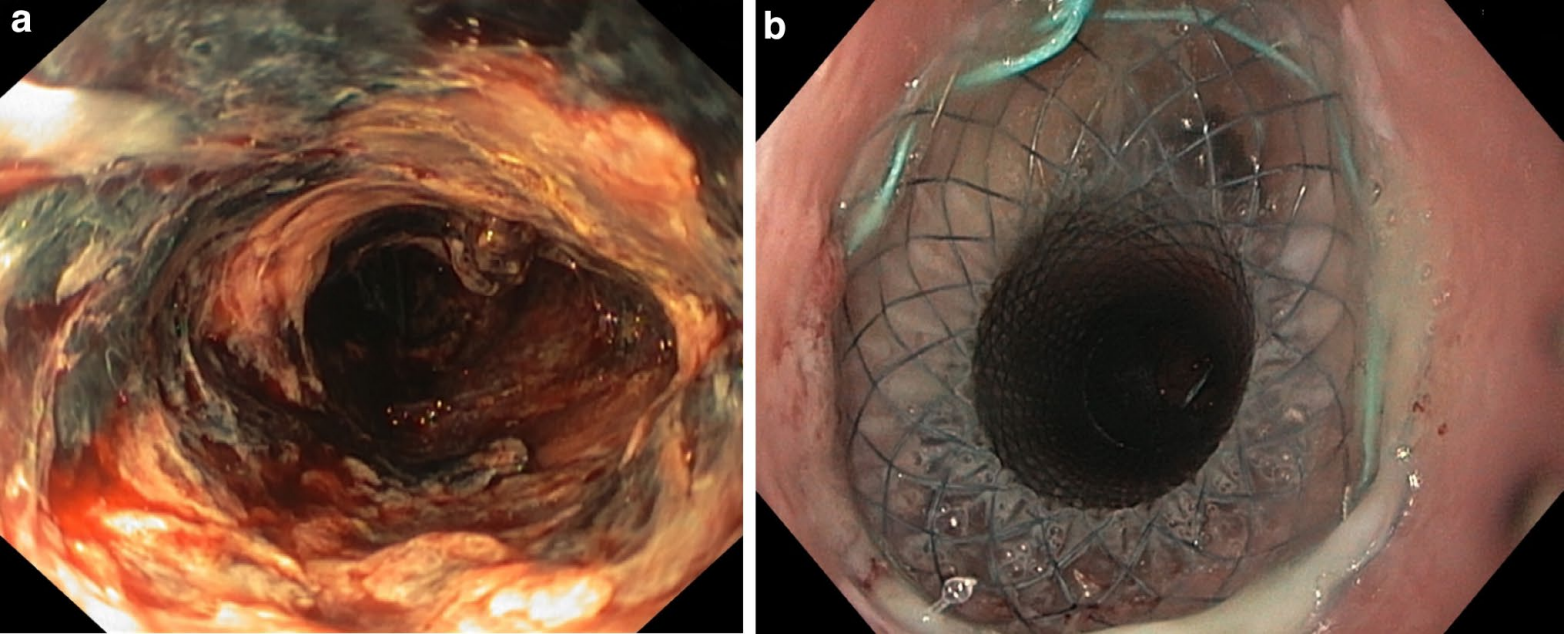
**a** Anastomotic leakage with necrotic tissue of the gastric sleeve after esophagectomy on day 7. **b** Following the first-line therapy with sponge, insertion of a SEMS stent on day 84 with subsequent successful closure of the defect

**Fig. 3:**
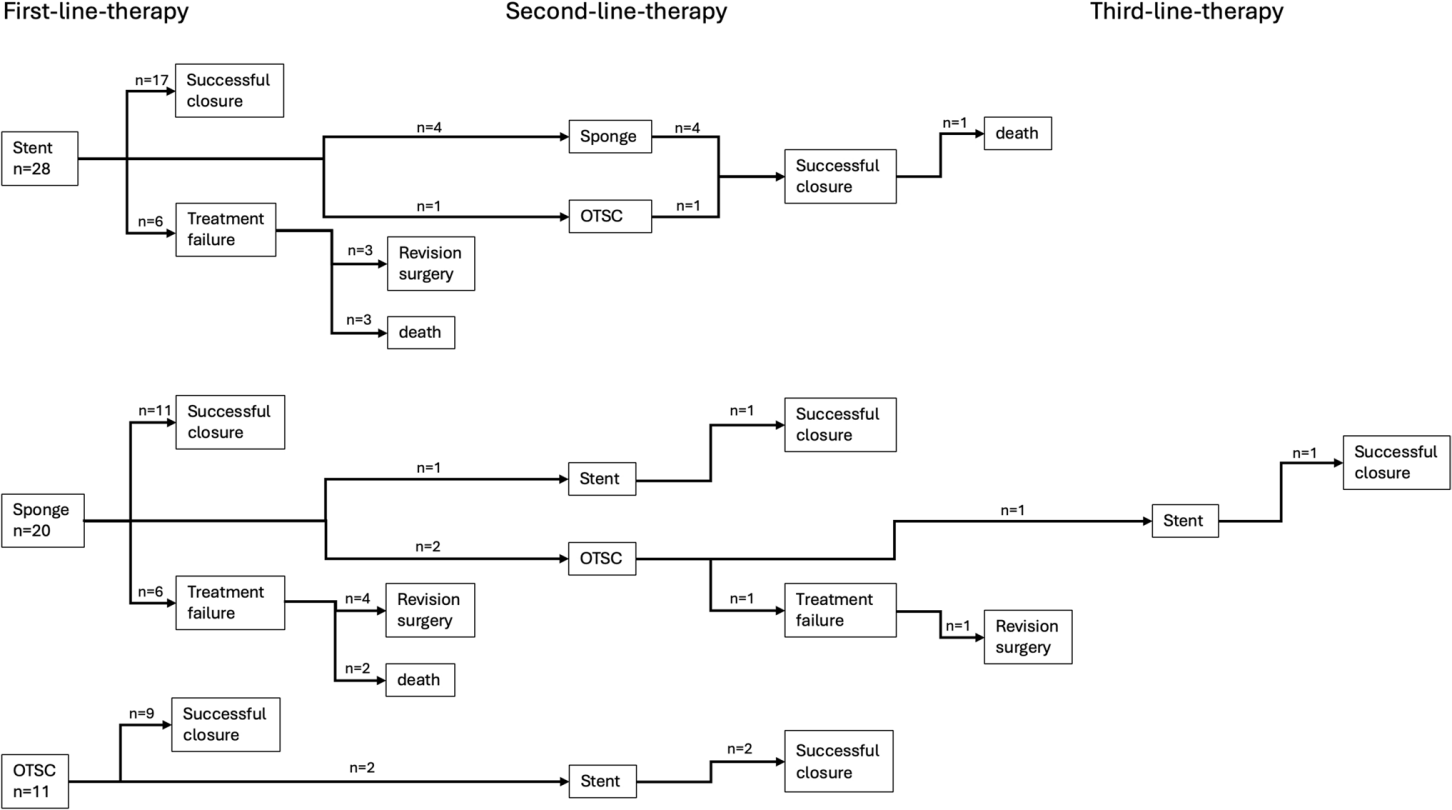
Flowchart illustrating the different treatment sequences of the included patients

Treatment modalities and outcome of the overall cohort and subgroups are displayed in Table 2. A median of 8 days (range 0–28) after surgery passed before the first endoscopic intervention was performed. Once started, treatment duration lasted for a median of 30 days (range 4–197) with a median in-hospital stay of 47 days (range 15–178). 40.7% of all patients received sponge treatment with a median of 3 (range 0–25) sponge changes. Overall, 54.2% of all patients received stent treatment, while 23.7% of patients were treated with an OTSC® Clip. Most patients were treated with only one treatment modality (either sponge or stent or OTSC® Clip, 83.1% of cases), while 15.3% of patients received a combination of two, and 1.7% a combination of three treatment modalities. Figure 3 illustrates the different treatment sequences. Successful endoscopic closure could be achieved in 78.0% of patients regardless of the treatment modality. The success rate for sponge treatment was 70.8%, for stent treatment 81.3%, and for OTSC® Clip treatment 92.9%. In-hospital mortality was 10.2%. Revision surgery was necessary in eight patients (13.6% of cases). Figures 1 and 2 each show an exemplary patient who received endoscopic therapy by insertion of a sponge or a SEMS stent.

**Table 2:** Treatment methods and outcome of the overall cohort and the subgroup of patients with and without endoscopic treatment success

Outcome parameter	All patients (*n* = 59)	Patients with endoscopic treatment success (*n* = 46)	Patients without endoscopic treatment success (*n* = 13)	*p*
Days until first endoscopic intervention, median (range)	8 (0–28)	8 (0–28)	6 (2–22)	0.324
Endoscopic treatment duration, days, median (range)	30 (4–197)	31 (4–197)	12 (4–70)	0.062
In-hospital stay, days, median (range)	47 (15–178)	49 (19–178)	44 (15–113)	0.345
Successful endoscopic closure, *n* (%)	46 (78.0)	46 (100.0)	0 (0.0)	
Sponge treatment, *n* (%)	24 (40.7)	17 (37.0)	7 (53.8)	0.274
Sponge changes, median (range)	3 (0–25)	6 (0–25)	3 (1–20)	0.480
Sponge success rate, *n* (%)	17 (70.8)	17 (100.0)	0 (0.0)	
Stent treatment, *n* (%)	32 (54.2)	26 (56.5)	6 (46.2)	0.508
Stent success rate, *n* (%)	26 (81.3)	26 (100.0)	0 (0.0)	
OTSC® treatment, *n* (%)	14 (23.7)	13 (28.3)	1 (7.7)	0.124
OTSC® success rate, *n* (%)	13 (92.9)	13 (100.0)	0 (0.0)	
Number of endoscopic treatment options				0.312
Single therapy only	49 (83.1)	37 (80.4)	12 (92.3)	
Combination of two	9 (15.3)	8 (17.4)	1 (7.7)	
Combination of three	1 (1.7)	1 (2.2)	0 (0.0)	
In-hospital mortality, *n* (%)	6 (10.2)	1 (2.2)	5 (38.5)	**< 0.001**
Need for anastomosis revision surgery, *n* (%)	8 (13.6)	0 (0.0)	8 (61.5)	**< 0.001**

Bold values are statistically significant (*p* < 0.05)

### Comparison of patients with endoscopic treatment success vs failure

Comparing the groups of patients with and without endoscopic treatment success, the treatment success group had a significantly lower ASA Score (*p* = 0.039) and a smaller defect size (*p* = 0.007), as shown in Table 1. The treatment success group also had a significantly lower in-hospital mortality (*p* < 0.001), as indicated in Table 2. No revision surgery was needed in the treatment success group compared to 61.5% in the group with endoscopic treatment failure (*p* < 0.001), see Table 2.

No significant differences were observed regarding age, sex, diabetes mellitus, obesity, UICC classification, neoadjuvant therapy, surgical approach, R-status after resection, reconstruction type, defect location, duration until first endoscopic intervention, endoscopic treatment duration, and in-hospital stay, as shown in Table 1 and 2.

#### Patients needing revision surgery

Of the eight patients receiving revision surgery, four initially underwent sponge therapy (see flowchart Fig. 3). Among these, two patients showed endoscopic evidence of gastric conduit necrosis. Another patient had previously undergone a complicated fundoplication at an external hospital, resulting in a large esophageal perforation that proved unmanageable by endoscopic means. One additional patient developed two further insufficiency sites following the initiation of sponge therapy, including an esophagotracheal fistula, which also proved endoscopically unmanageable over the course of treatment. Of the eight patients receiving revision surgery, one patient initially received sponge therapy followed by placement of an OTSC clip (see flowchart Fig. 3). Nevertheless, a persistent insufficiency behind the clip remained refractory to endoscopic therapy, accompanied by putrid secretion and pleural empyema requiring rethoracotomy. Three of the eight patients who required revision surgery had initially received primary stent therapy (see flowchart Fig. 3). In all three cases, however, persistent purulent secretion was observed via the inserted drains, ultimately necessitating revision of the anastomosis and surgical evacuation of the empyema.

#### Binary univariate and multivariate logistic regression model

Comparing the groups of patients with and without endoscopic treatment success in an univariate logistic regression model, the treatment success group had a significantly lower ASA Score (95% CI 0.120–0.891, OR 0.327, *p* = 0.029) and a smaller defect size (≤ 1 cm vs > 1 cm, 95% CI 1.492–37.978, OR 7.526, *p* = 0.015). The treatment success group also exhibited a significantly lower in-hospital mortality (95% CI 0.004–0.346, OR 0.036 *p* = 0.004), as shown in Table 3.

**Table 3:** Binary univariate logistical regression model, dependent variable: endoscopic treatment success

Variable	Odds ratio	95% confidence interval		*p*
Age, y	0.98	0.92	1.06	0.661
Male sex	0.37	0.10	1.32	0.126
ASA Score (I–IV)	0.33	0.12	0.89	**0.029**
Diabetes mellitus	1.03	0.24	4.49	0.968
Obesity	0.93	0.17	5.06	0.928
Smoking	0.94	0.25	3.58	0.927
UICC classification	0.92	0.64	1.32	0.65
Neoadjuvant therapy	0.35	0.07	1.83	00.213
Location of the defect	0.88	0.78	1.01	0.065
Residual tumor status (R-status) after resection	0.55	0.05	6.11	0.629
Defect size ≤ 1 cm	7.53	1.49	37.98	**0.015**
Days until first endoscopic intervention	1.00	0.89	1.12	0.971
Endoscopic treatment duration, days	1.03	0.99	1.07	0.13
In-hospital stay, days	1.01	0.98	1.03	0.529
Sponge treatment	0.50	0.15	1.74	0.278
Sponge changes	1.07	0.91	1.26	0.432
Stent treatment	1.52	0.44	5.22	0.509
OTSC® treatment	4.73	0.56	40.12	0.155
Number of endoscopic treatment options	2.85	0.36	22.64	0.322
In-hospital mortality	0.04	0.01	0.35	**0.004**
Need for revision surgery	N/A	N/A	N/A	N/A
Major bleeding	N/A	N/A	N/A	N/A
Fistula	0.12	0.01	1.47	0.098
Stenosis	N/A	N/A	N/A	N/A
Stent dislocation	1.53	0.29	8.05	0.617
In-grown stent	N/A	N/A	N/A	N/A
Sepsis	0.62	0.18	2.17	0.456
Mediastinitis	N/A	N/A	N/A	N/A
Pleural empyema	1.05	0.24	4.50	0.95

Bold values are statistically significant (*p* < 0.05)

N/A: Could not be calculated meaningfully since the event did not occur in one of the two groups

Considering the sample size, the variables defect size and ASA Score were analyzed in a multivariate logistic regression model, as presented in Table 4. With endoscopic treatment success as the dependent variable both a higher ASA Score (95% CI 0.092–0.907, OR 0.289, *p* = 0.033) and a larger defect size (95% CI 1.607–48.761, OR 8.852, *p* = 0.012) were independent risk factors for treatment failure.

**Table 4:** Binary multivariate logistical regression model, dependent variable: endoscopic treatment success

Variable	Odds ratio	95% confidence interval		*p*
ASA score (I–IV)	.29	0.09	0.91	**0.033**
Defect size ≤ 1 cm	8.85	1.61	48.76	**0.012**

Bold values are statistically significant (*p* < 0.05)

#### Complications

The most common complication observed was sepsis which occurred in 22 of 59 patients (37.3%, Table 5). Mediastinitis occurred in 2 of 59 patients (3.4%) and pleural empyema in 14 of 59 cases (8.3%). The development of fistulas (tracheoesophageal, bronchoesophageal, lymphatic fistula) occurred in 3 of 59 patients (5.1% of cases). A total of 32 patients received a stent placement, with 12 endoscopy reports noting a stent dislocation in the further course of treatment. However, these dislocations were typically minor and did not require correction. In one case stent fixation via OTSC® Stentfix (Ovesco) was necessary. Three endoscopy reports noted an “in-grown” stent upon stent removal. Still, stent removal caused no further complications in these cases. One patient who had received a stent placement and another who received a sponge treatment developed a subsequent stricture at the site of anastomosis requiring dilatation in the further course of treatment. Additionally, we observed a major bleeding event due to an aorto-esophageal fistula. The patient in question was a 57-year-old woman who underwent Ivor Lewis esophagectomy for an AEG II tumor of the esophagus. On postoperative day 13, an esophageal leak was identified endoscopically, prompting immediate initiation of endoluminal vacuum therapy (EVT) using a sponge system. Eighteen days after the start of sponge therapy, an emergency upper endoscopy was performed due to suspected upper gastrointestinal bleeding, revealing active bleeding presumably originating from the area of the anastomotic insufficiency. A Sengstaken tube was placed, followed by an emergency CT angiography. The imaging revealed contrast extravasation from the descending aorta at the level of the carina, raising a strong suspicion of an erosive hemorrhage from the descending aorta. The patient subsequently developed hemorrhagic shock requiring cardiopulmonary resuscitation, which led to return of spontaneous circulation (ROSC) after 20 min. Emergency surgical intervention was performed immediately thereafter, confirming an erosive hemorrhage due to an aorto-esophageal fistula. The patient underwent thoracic endovascular aortic repair (TEVAR), which successfully controlled the bleeding, and was subsequently transferred to the intensive care unit. EVT was resumed in the further course and, after successful healing of the insufficiency, the sponge system was removed on day 28 following its initial placement. The patient was discharged to outpatient care after a total hospital stay of 66 days.

**Table 5:** Observed complications in the overall cohort

Complication	All patients (*n* = 59)	Endoscopic treatment success (*n* = 46)	No endoscopic treatment success (*n* = 13)	*p*^§^	Sponge treatment (*n* = 24)	Stent treatment (*n* = 32)	OTSC® clip (*n* = 14)
Major bleeding, *n* (%)	1 (1.7)*	1 (2.2)	0 (0.0)	0.592	1 (4.2)	0 (0.0)	0 (0.0)
Fistula, *n* (%)	3 (5.1)	1 (2.2)	2 (15.4)	0.056	2 (8.3)	0 (0.0)	1 (7.1)
Stenosis, *n* (%)	2 (3.4)	2 (4.3)	0 (0.0)	0.444	1 (4.2)	0 (0.0)	1 (7.1)
Stent dislocation, *n* (%)	12 (20.3)	10 (21.7)	2 (15.4)	0.615	3 (12.5)	12 (37.5)	1 (7.1)
In-grown stent, *n* (%)	3 (5.1)	3 (6.5)	0 (0.0)	0.345	0 (0.0)	3 (9.4)	0 (0.0)
Sepsis, *n* (%)	22 (37.3)	16 (43.8)	6 (46.2)	0.454	5 (20.8)	15 (46.9)	6 (42.9)
Mediastinitis, *n* (%)	2 (3.4)	2 (4.3)	0 (0.0)	0.444	0 (0.0)	2 (6.3)	0 (0.0)
Pleural empyema, *n* (%)	14 (8.3)	11 (23.9)	3 (23.1)	0.950	1 (4.2)	11 (34.4)	5 (35.7)

*Aorto-esophageal fistula with erosion bleeding after sponge therapy. Patient survived after emergency surgery

^§^Chi-Square Test, dependent variable: endoscopic treatment success

## Discussion

In our study, we retrospectively analyzed data from a cohort of patients with postoperative anastomotic leakage following esophagectomy and consecutive endoscopic treatment. Anastomotic leakage is still the most feared complication after esophagectomy, associated with high mortality and morbidity, despite new surgical approaches (robotic-assisted minimally invasive esophagectomy) promising a better outcome [14]. In recent years, new endoscopic approaches have been developed to effectively treat anastomotic leakage. These particularly include endoscopic vacuum therapy, which now has become an established and wildly used treatment option. However, most of the studies published to date are single-center studies with low case numbers making it difficult to compare outcome parameters. To improve the reliability of outcome data, we pooled cases from two centers.

The overall endoscopic closure rate in our study was 78.0% (Sponge 70.8%, Stent 81.3%, OTSC® 92.9%). The sponge closure rate was at the lower end of the published data, while stent closure was well within the published success rate [11, 12, 15]. The fact that the participating hospitals are tertiary centers with referrals of severe cases, probably plays an important role in the outcomes. We demonstrate that small defects of ≤ 1 cm can be effectively managed endoscopically, with a closure rate of 93% (26 out of 28). The two unsuccessful cases showed insufficient drainage of the cavity and were later treated successfully with surgical revision. Accordingly, in cases of endoscopic treatment failure close collaboration between endoscopists and surgeons is warranted to provide patients with the most suitable therapeutic option and avoid treatment delay.

The mortality rate in our study (10.2%, or 6 out of 59 cases) is in line with published data. Bludau et al. reported a mortality rate of 9 out of 59 patients (15.3%) with postoperative leaks [8], whereas Laukoetter et al. reported an in-hospital-mortality of 9.6% in a total of 52 patients [7].

In our study, we aimed to identify possible risk factors for an unfavorable outcome. We found that patients with a higher ASA Score and thus more severe comorbidities had a higher risk for endoscopic treatment failure. Comorbidities like age, diabetes, obesity, smoking, and immunocompromising conditions such as malignancies have previously been identified as relevant systemic risk factors affecting wound healing [16]. For instance, patients with diabetes often exhibit reduced levels of vascular endothelial growth factor (VEGF)—a key proangiogenic factor—which compromises the process of local angiogenesis [17]. Nicotine is also believed to disrupt oxygen supply by promoting tissue ischemia via vasoconstrictive effects [18]. In addition to its effect on tissue, smoking increases the risk of developing atherosclerosis and chronic obstructive pulmonary disease—both conditions that can further reduce tissue oxygen supply [19, 20].

A defect size of over 1 cm led to a significantly higher failure rate in this study. While it seems conclusive that larger leaks may be at more risk not to heal, this study is the first to our knowledge to show a significant correlation. Sponge treatment becomes more difficult with a defect size of > 3 cm due to the limitation of the sponge size that can be passed down the esophagus [21]. Conversely, numerous publications documented successful healing of larger defects with endosponge therapy, suggesting that while defect size is likely a relevant factor for the success of endoscopic treatment, it is not the sole determinant [22, 23]. Other predictive factors associated with the success of sponge or SEMS therapy could not be identified although we considered variables, such as the time until start of endoscopic therapy, the duration of therapy, location of the defect, and treatment complications.

The time until detection of an esophageal leakage following esophageal surgery appears to be a critical factor in determining the subsequent clinical course. A commonly used surrogate parameter for this is the postoperative day on which the first endoscopic intervention takes place. In this study, the median time for the initial endoscopic procedure was the eighth postoperative day. Similarly, in a study involving 157 patients who underwent robotic-assisted minimally invasive esophagectomy at a high-volume center, anastomotic leakage was observed in 21 patients, with the diagnosis also typically occurring on postoperative day 8 [14]. This finding further emphasizes the critical importance of early diagnosis of anastomotic leakage. The sooner the condition is identified, the smaller the resulting defect is likely to be—and as demonstrated in this study, smaller defects are more amenable to successful endoscopic closure. For clinicians managing these patients, this underscores the need for a low threshold to perform early endoscopic evaluation of the anastomosis when there is any clinical suspicion of leakage, enabling prompt and effective intervention. An exception to this approach arises in cases of early anastomotic leakage combined with conduit necrosis, where surgical revision is typically the preferred course of treatment [24].

Another risk factor that has been described is the presence of mediastinitis after the initiation of endoscopic therapy, even though the statistical evaluation in this study did not reflect this association [25]. The development of mediastinitis due to an anastomotic leakage is a feared complication and associated with high morbidity and mortality. As soon as the first signs appear during the postoperative period prompt and effective management is warranted, ensuring adequate external drainage and preventing any additional contamination from gastrointestinal contents [26]. A higher endoscopic treatment failure rate in these patients can therefore be explained by an insufficient drainage of the affected site. Neoadjuvant treatment has also previously been identified as a risk factor for anastomotic complications [27, 28]. In another study neoadjuvant therapy was associated with longer endoscopic treatment duration [29], although this was not reflected in our data.

Sepsis was the most common complication in our study occurring in 22 out of 59 patients (37%). A recently published registry study on sponge therapy reported a sepsis rate of approximately 10% [25]. Another study identified mediastinitis as their most common complication, occurring in 43% of their cases [19]. Surgical treatment of esophageal cancer by esophagectomy with reconstruction is associated with a high morbidity rate. Bartels et al. reported a postoperative complication rate of approximately 20% in a cohort of 944 consecutive patients operated on over a period of 18 years. The overwhelming majority of these complications (approx. 85%) were surgery-related, with 70% being sepsis related. Sepsis almost always resulted from an anastomotic leakage while complications associated with ICU therapy (e.g., ventilator-associated pneumonia) played a minor role [26].

Postinterventional anastomotic stenosis was observed in 3.4% of cases after successful endoscopic treatment in line with previous publications [25, 30, 31]. All strictures were effectively managed with multiple sessions of balloon dilation. All SEMS dislocations were minor and could be managed by simple repositioning. In one case stent fixation via OTSC® Stentfix was necessary. The migration of SEMS remains a common complication following their insertion. According to a meta-analysis, esophageal SEMS showed a pooled migration rate of 31.5% [32]. Over-the-scope clip fixation is an effective method to prevent displacement by anchoring the SEMS [33].

It is noteworthy that we observed the development of an aorto-esophageal fistula in one patient, resulting in life-threatening erosion bleeding after sponge therapy. This represents a very rare but serious complication of endoscopic vacuum therapy which has been described in the literature [34]. Our patient survived following emergency surgery. Laukoetter et al. also reported about two patients with fatal hemorrhage after endoscopic therapy for late anastomotic leaks. Although it could not be proven that an erosion bleeding was the cause of the hemorrhage in these cases, they recommended performing a thoracic CAT scan immediately before or after the initial placement of a sponge to exclude close proximity to cardiovascular structures [7].

Strengths of our study include its relatively large patient cohort compared to the existing literature [15]. However, a limitation of our study is its retrospective design. Thus, information bias can be a possible confounder. Since some of the included cases date further back in time, we refrained from a direct comparison of the treatment modalities, as sponge therapy was simply not available or not yet introduced at the time. Accordingly, the earliest cases were treated exclusively with stents, while the first documented sponge placement in our cohort occurred in 2011. In recent years, several innovative and promising endoscopic closure techniques have been developed, including the VAC-Stent [13], the X-tack™ System [35], and the SutuArt System [36]. To effectively evaluate and compare these emerging methods in future, a robust data foundation on established closure techniques is essential. The findings of this study strengthen the existing evidence for current standard therapies and provide a solid basis for the scientific assessment of these novel approaches.

### Study conclusion

Taken together, our study demonstrates that small defects (≤ 1 cm) can almost always be managed successfully with endoscopic treatment, effectively avoiding revision surgery and being associated with low mortality. We could also show that the severity of a patient’s comorbidities influences treatment outcomes. Potential severe risks of endoscopic vacuum therapy, including fatal erosion bleedings, should be considered initiating therapy. Future prospective trials are needed to validate our results and further refine treatment strategies.
